# Negative association of endothelial nitric oxide gene polymorphism with hypertension in Turkish patients: effect of ecNOS polymorphism on left ventricular hypertrophy

**DOI:** 10.1186/1476-7120-4-33

**Published:** 2006-08-21

**Authors:** Ayhan Olcay, C Gokhan Ekmekci, Ugur Ozbek, Murat Sezer, Cem Barcin, Erol Arslan, Bilal Boztosun, Yilmaz Nisanci

**Affiliations:** 1 Department of Cardiology, Istanbul School of Medicine, Istanbul, Turkey; 2 Department of Genetics, Istanbul School of Medicine, Institute for Experimental Medicine, Istanbul, Turkey; 3 Department of Cardiology, Ankara Gendarmerie Hospital, Ankara, Turkey; 4 Department of Cardiology, Kosuyolu Heart Education and Research Hospital, Istanbul, Turkey

## Abstract

**Background:**

Endothelial nitric oxide synthase produces nitric oxide which is involved in many physiologic regulatory functions. Variable number of tandem repeats in intron 4 of endothelial nitric oxide synthase gene are reported to be associated with blood pressure regulation. Nitric oxide is involved in regulation of cardiomyocyte genes but it is not known If endothelial nitric oxide synthase 4 gene polymorphisms are related with left ventricular hypertrophy. We studied endothelial nitric oxide synthase 4a/b allele status in hypertensive and normotensive patients and echocardiographic parameters in a subgroup of hypertensive group.

**Methods:**

We performed a case-control study involving 110 Turkish hypertensive patients and 87 controls. All subjects were genotyped for endothelial nitric oxide synthase 4a/b polymorphism. Echocardiographic measurements were obtained in 94 of the hypertensive patients.

**Results:**

Endothelial nitric oxide synthase 4a/b genotype frequencies were 6.4%, 23.6%, 70% in hypertensives and 1.1%, 18.4%, 80.5% in controls for a/a, a/b, b/b, respectively. Left ventricular dimensions, mass and diastolic indices were not different across endothelial nitric oxide synthase 4 genotypes. Patients with 4a/a genotype had higher interventricular septal thickness than the other group; 14.83(1.6), 11.91(1.51), 12.21(1.56) for a/a, a/b, b/b, respectively and p = 0.0001.

**Conclusion:**

Endothelial nitric oxide synthase 4a/b gene polymorphism is not associated with hypertension in Turkish patients. 4a/a genotype was associated with higher interventricular septal thickness in hypertensive patients.

## Background

Endothelial nitric oxide synthase (ecNOS; NOS3) produces nitric oxide (NO) from L-arginine. NO has diverse physiologic regulatory functions and is involved in smooth muscle relaxation, inhibition of platelet aggregation, immune regulation, neurotransmission and blood pressure regulation [1-3]. NO deficiency induces vascular and cardiac hypertrophy and inhibition of ecNOS reduces coronary flow and raises blood pressure [4,5]. NO regulates expression of genes in cardiac myocytes that are important in cardiac hypertrophy and failure. In cDNA expression array analysis 10 genes out of the 1176 endothelial genes were consistently up- or downregulated by NO [6]. ecNOS is encoded by a gene located on chromosome 7q35–36, expressed in endothelium. Some ecNOS alleles affect nitric oxide levels and cardiovascular system. There are two alleles identified in intron 4 of the ecNOS gene. The larger allele, 4b, consists of five tandem 27-bp repeats (variable number of tandem repeats-VNTR) and smaller one, 4a, has four repeats [7,8]. An association of the 4a allele of the ecNOS gene with coronary artery disease and renal disease was reported [8-11]. The intron 4 VNTR polymorphism was associated with altered plasma NO levels and variations in plasma nitrite and nitrate levels [9,12]. However, conflicting data have appeared in the literature concerning the association between this variant and hypertension. In fact, the intron 4 VNTR polymorphisms were associated with hypertension in one Japanese study but other studies found no association [13-15]. Interethnic differences in NO-mediated vasodilation may result from disproportionate distribution of ecNOS variants in ethnic groups. Interethnic differences in ecNOS variants may result in a higher incidence and severity of hypertension in African-Americans. Genetic determinants underlying interethnic differences in drug response can help to predict the drug effects and improve therapy [16-18]. To the best of our knowledge there is no data about ecNOS4a/b polymorphism in Turkish hypertensive patients.

In our study we examined ecNOS gene intron 4 VNRT polymorphism in hypertensive and normotensive Turkish patients. Echocardiographic examinations were obtained in a subgroup of hypertensive patients and relationship between echocardiographic left ventricular indices and ecNOS intron 4 VNRT polymorphism determined.

## Methods

### Study population

A total of 110 hypertensive patients who were under drug treatment were included into the study from Istanbul School of Medicine Cardiology Department. Blood samples were obtained from 90 healthy individuals applying to outpatient clinic of internal medicine with nonspecific complaints. All of the hypertensive patients were on one or more antihypertensive drugs. Patients were all in mild to moderate hypertension group. Hypertension control was assessed by the cardiologist in the outpatient clinic and all patients were reported to have a well controlled hypertension. DNA for analysis was not available for three subjects in control group. Echocardiographic measurements were obtained in 94 hypertensive patients. Patients who had rheumatic valvular disease, myocardial infarction, renal disease and secondary hypertension were not enrolled into the study. Ethics committee approval and informed consent was obtained from all patients. Anthropometric measurements and clinical parameters were determined by a questionnaire based study. Echocardiographic and genotype data were obtained by independent observers unaware of the other test results.

### Genotyping

#### Detection of eNOS polymorphism

ecNOS4a/b genotypes were determined by polymerase chain reaction (PCR) using oligonucleotide primers (sense: 5'-AGGCCCTATGGTAGTGCCTTT-3' ; antisense, 5'-TCTCTTAGTGCTGTGGTCAT-3' Prizma Laboratory Products Industry and Trade Co. LTD., Istanbul, TR) that flank the region of the 27 bp direct repeat in intron 4 as described previously with minor modifications [11]. Reactions were performed in a total volume of 50 μL containing 500 ng genomic DNA, 10 pmol of each primer, 0.2 mM dNTP, 0.5 U Taq DNA polymerase, 5 μL PCR buffer (500 mmol/L KCL, 100 mmol tri-hidroksimetil-aminometan chloride and 0.8% Nonidet P40). The thermocycling (Perkin Elmer Cetus, DNA Thermal Cycler 480, USA and Eppendorf Mastercycler Personal 5332, Germany) procedure consisted of initial denaturation at 94° for 5 min, 35 cycles of denaturation for 94° for 1 min, annealing at 55° for 1 min, extension at 72° for 1 min. The PCR products were analyzed using 3% agarose gel electrophoresis and visualized by ethidium bromide staining. The large allele, ecNOS4b, contains 5 tandem 27 bp repeat and the smaller allele, ecNOS4a, contains 4 repeats. The size of the PCR products were 393 bp and 420 bp for the ecNOS4a and ecNOS4b alleles, respectively.

### Echocardiography

All patients were studied by M-mode echocardiography to determine left ventricular size (left ventricular end diastolic diameter- LVED, left ventricular end systolic diameter-LVES, interventricular septum-IVS and posterior wall thickness-PW) by three expert sonographers with two recorders (Vingmed System V and Vivid III, General Electric). Pulsed doppler velocimetry was used to determine peak early (E) and late atrial (A) diastolic transmitral velocities. Penn convention criteria were applied for measurement of left ventricular dimension and calculation of LV mass-LVM [19]. Left ventricular mass index-LVMI was calculated by indexation of LVM to body surface area. LV ejection fraction was calculated according to the modified Simpson formula [20].

### Statistical analysis

Data were analyzed using SPSS for Windows release 7.5.1. Percentages were compared by X^2 ^analysis. According to ecNOS4a/b allele status, continuous data were compared with the use of ANOVA. Effects of NOS4a/b allele status on LV mass index (obtained by indexation of LVM to body surface area), LVED/m2 (LVED indexed to body surface area), LVES/m2 (LVES indexed to body surface area) LV ejection fraction (EFM), fractional shortening (FS), diastolic E and A velocities were examined with multiple linear regression analysis after adjustment for age, gender, body mass index, atrial fibrillation (AF), diabetes mellitus (DM) and hypertension treatment status (HTTx). LV hypertrophy (LVH) was defined as an LV mass index of > 134 g/m^2 ^in men or > 110 g/m^2 ^in women. Left ventricular hypertrophy (absence or presence) was analyzed as a categorical variable by logistic regression. Continuous data are summarized as mean ± SD or as median. All tests were two sided, and a P value of less than 0.05 was considered to indicate statistical significance

## Results

### Genotype frequencies

We genotyped 110 hypertensive patients and 87 healthy controls for the ecNOS 4a/b gene polymorphism. Clinical characteristics of the study group are summerized in Table 1. There was no significant differences between groups with respect to sex distribution and presence of diabetes mellitus. Control group subjects were younger than hypertensive patients, 40.53(15.83) vs 59.41(10.14) and p = 0.0001. Smoking was more frequent in control group, 34.5 vs 21.8 and p = 0.048.

**Table 1:** Clinical characteristics of the study group

Characteristic	HT n = 110	NT n = 87	P
Age, year	59.41(10.14)	40.53(15.83)	0.0001
Sex, M/F %	39.1/60.9	47.8/52.2	0.21
Smoking, %	21.8	34.5	0.048
Diabetes Mellitus, %	16.4	5.6	0.17

HT: hypertensive subjects, NT: healthy controls

The distribution of genotypes and allele frequencies were compared between patients and controls in Table 2. The genotype frequencies were in agreement with Hardy-Weinberg equilibrium. ecNOS4a/b genotype frequencies in hypertensives were 6.4%, 23.6%, 70% for aa, ab, bb, respectively and 1.1%, 18.4%, 80.5% for aa, ab, bb, respectively in control group. Genotype frequencies were not statistically different between groups (X^2 ^= 4.59, p = 0.10).

**Table 2:** ecNOS4a/b genotype distribution in hypertensive patients and controls

ecNOS4a/b genotypes	HT	NT
a/a, n, (%)	7 (6.4)	1 (1.1)
a/b, n, (%)	26 (23.6)	16 (18.4)
b/b, n, (%)	77 (70)	70 (80.5)
Total, n, (%)	110 (100)	87 (100)
X^2 ^= 4.59 P = 0.10		

HT: hypertensive subjects, NT: healthy controls

### Echocardiographic measurements

Echocardiographic examinations were obtained from 94 patients in hypertensive group. Clinical characteristics of the hypertensive subgroup of patients according to ecNOS4 allele status are listed in Table 3. Age, sex, prevalence of smoking, diabetes mellitus, atrial fibrillation, duration of hypertension were not statistically different between three groups. All patients were receiving antihypertensive therapy and none of the patients consumed alcohol.

**Table 3:** Clinical characteristics of hypertensive patients included in echocardiographic substudy

Characteristic	ecNOS4a/b polymorphism			
	a/a n = 6	a/b n = 22	b/b n = 66	p

Sex, M/F	1/5	8/14	26/40	X^2 ^= 1.22 p = 0.54
Age, y	59(10.06)	62.14(9.21)	58.28 (10.639)	0.30
DM, %	33.3	18.2	15.2	0.51
Smoking, %	16.6	22.7	19.7	0.93
Alcohol, %	0	0	0	--
AF, %	33.3	9.1	6.1	0.70
HT age	5.83(2.04)	6.59(3.23)	9.02(7.4)	0.20
HT Tx, %	100	100	100	--

DM: diabetes mellitus, AF: atrial fibrillation, HT age: duration of hypertension, HT Tx: treatment for hypertension.

Echocardiographic measurements are listed in Table 4. Left ventricular end systolic and end diastolic dimensions indexed to body surface area, posterior wall thickness, left ventricular ejection fraction, fractional shortening, left ventricular mass index, early diastolic transmitral peak velocity (E), late diastolic transmitral peak velocity and left atrial sizes showed no statistically significant differences across aa, ab or bb genotypes. Interventricular septal thickness was 14.83(1.6), 11.91(1.51), 12.21(1.56) for aa, ab, bb, respectively and p = 0.0001. Patients with 4a/a genotype had higher septal thickness than the other groups and difference was statistically significant. Left ventricular hypertrophy was present in 75.3% of the study population. Left ventricular hypertropy percentages were not statistically different across ecNOS4 genotypes.

**Table 4:** Echocardiographic measurements and ecNOS4a/b genotypes in hypertensive patients included in echocardiographic substudy

Variable	ecNOS4a/b polymorphism			
	a/a	a/b	b/b	p

LVEDD	27.22(2.93)	27.72(4.53)	27.65(5.03)	0.93
LVESD	17.44(3.25)	18.61(5.18)	18.32(5.26)	0.89
IVS, mm	14.83(1.6)	11.91(1.51)	12.21(1.56)	0.0001
PW, mm	12.17(1.94)	10.95(1.43)	10.74(1.51)	0.92
LV EF, %	65.33(8.45)	60.82(14.61)	61.97(14.33)	0.78
LV FS, %	36.17(7.11)	33.14(10.22)	34.06(9.69)	0.78
LVMI, g/m^2^	173.5(27.29)	141.77(37.31)	151.78(56.32)	0.39
LVH, %	100	77.3	72.3	X2 = 2.23 p = 0.31
E, cm/s	57(26.06)	64.53(21.06)	72.05(24.3)	0.33
A, cm/s	96(16.64)	77.94(13.75)	80.93(26.77)	0.49
LA size, mm	43(12.81)	39.5(6.26)	37.26(5.42)	0.56

LVEDD: left ventricular end-diastolic dimension mm/m2, LVESD: left ventricular end-systolic dimension mm/m2, IVS: interventricular septal thickness mm/m2, PW: posterior wall thickness mm/m2, LV FS: left ventricular fractional shortening, LVMI: left ventricular mass index (Penn formula), E: early diastolic transmitral peak velocity, A: late diastolic transmitral peak velocity, LA: left atrium size mm.

## Discussion

The results of our study indicate that ecNOS4a/b polymorphism is not associated with with hypertension, left ventricular dimension or mass but is associated with interventricular septal thickness. To our knowledge there was no previous study about relation of ecNOS4a/b polymorphism with hypertension in Turkish population. Most of the previous studies about ecNOS4a/b polymorphism and hypertension in literature are negative association studies and we examined whether there is any positive association in our study population [10,15]. A previous Turkish study showed that patients with myocardial infarction had higher frequency of a/a genotype, 4.3% [21]. In the study genotype distribution in normal population was 0.6% for a/a, 18% for a/b and 81.4% for the b/b genotype. There was no statistically significant difference in ecNOS4a/b allele percentages between cases and controls. The defect in NO production in hypertension could involve a gene other than ecNOS4. In fact, discrepancies in association studies may also result from consideration limited to only one polymorphism rather than combinations of polymorphisms. In particular, recent data suggest that individual eNOS genotypes are not reliable markers of risk for developing hypertension, but study of eNOS haplotypes may provide much more appropriate information [22,23].

In 94 of the hypertensive patients we obtained echocardiographic measurements to see whether any subgroup of ecNOS4a/b polymorphism is associated with left ventricular dimensions or mass. Although there are previous animal studies about effect of NO on vascular and cardiac myocytes, relation of ecNOS4a/b polymorphism with left ventricular hypertrophy and function in hypertension was not examined in humans previously [13]. In a previous human study ecNOS Glu298Asp polymorphism was not found to be related with blood pressure, left ventricular mass or carotid intima media thickness [24]. In another study three polymorphisms including -922A>G, intron 4VNTR, and Glu298Asp of the eNOS gene were investigated and no associations between single polymorphisms or haplotypes of the ecNOS gene and systolic blood pressure or left ventricular mass were found [25].

In our hypertensive subgroup of patients left ventricular dimensions, left ventricular mass index and left ventricular diastolic indices were not different in ecNOS4a/b subgroups. On the other hand interventricular septal thickness was higher in a/a genotype; 14.83(1.69) for a/a, 11.91(1.51) for a/b, 12.21(1.56) for bb and p = 0.0001. Although all patients were under hypertension treatment, septal hypertrophy in a/a genotypes may be due to higher susceptibility of those patients for hypertrophy. Relation of ecNOS4a/b polymorphism and left ventricular hypertrophy needs to be determined in untreated hypertensive patients to see if there is a similar association in untreated patients.

Limitations of our study are that all study patients were treated with different drugs and a detailed subgroup analysis can not be done due to small sample size. Echocardiographic measurements may be suboptimal when compared to toher methods like magnetic resonance imaging.

In conclusion ecNOS4a/b polymorphism is not associated with hypertension in Turkish population. Echocardiographic left ventricular dimensions and left ventricular mass are not associated with ecNOS4a/b polymorphism in treated hypertensive patients. Interventricular septal thickness is increased in patients with ecNOS4 a/a genotype. Relation of ecNOS4a/b genotype and left ventricular hypertrophy needs to be determined in untreated hypertensive patients. If genetic factors determining left ventricular hypertrophy and dilatation in hypertensive patients are determined, those patients under greater risk can be treated more aggressively and with drugs suitable to genetic markers.

## Competing interests

The author(s) declare that they have no competing interests.

## Authors' contributions

AO participated in design and coordination of the study, statistical analysis, in sequence alignment and drafted the manuscript. CGE carried out the molecular genetic studies, participated in the sequence alignment and drafted the manuscript. UO carried out the molecular genetic studies, participated in the sequence alignment and drafted the manuscript.

MS carried out patient enrollment, statistical analysis and manuscript preparation. CB participated in design of the study, statistical analysis and manuscript preparation. EA participated in design of the study, statistical analysis and manuscript preparation. BB patient enrollment, statistical analysis and final revisions of the manuscript. YN participated in design and coordination of the study, statistical analysis and drafted the manuscript
